# Real-World Outcomes of Adjuvant Paclitaxel and Trastuzumab Therapy in Lymph Node-Negative, HER2-Positive Early-Stage Breast Cancer: A Multicenter Retrospective Data Analysis

**DOI:** 10.3390/cancers17142271

**Published:** 2025-07-08

**Authors:** Buket Şahin Çelik, Pınar Peker, Ender Eren Özçelik, Ömer Faruk Kuzu, Erhan Gökmen, Gül Başaran, Türkkan Evrensel

**Affiliations:** 1 Ege Üniversitesi Tıp Fakültesi Hastanesi, İzmir 35100, Türkiye; pnnarrrr@gmail.com (P.P.); erhan.gokmen@ege.edu.tr (E.G.); 2 Bursa Uludağ Üniversitesi Tıp Fakültesi Hastanesi, Bursa 16059, Türkiye; endereren@uludag.edu.tr (E.E.Ö.); evrensel@uludag.edu.tr (T.E.); 3 Gülhane Eğitim ve Araştırma Hastanesi, Ankara 06010, Türkiye; drfarukkuzu@gmail.com; 4 Acıbadem Altunizade Hastanesi, İstanbul 34662, Türkiye; gul.basaran@acibadem.edu.tr

**Keywords:** HER2-positive breast cancer, adjuvant therapy, trastuzumab, paclitaxel, recurrence-free survival, real-world data

## Abstract

**Background**: Approximately 15–20% of early-stage breast cancers overexpress HER2, which is associated with an increased risk of recurrence. Although adjuvant anti-HER2 therapies have significantly improved patient outcomes, the optimal treatment strategy remains uncertain, particularly for patients with small, lymph node-negative tumors, where concerns about potential overtreatment and toxicity persist. The objective of this study was to evaluate overall survival (OS), recurrence-free survival (RFS), and treatment-related neuropathy in patients with early-stage HER2-positive breast cancer treated with adjuvant trastuzumab and paclitaxel. **Methods**: A total of 129 patients, aged 18 to 75 years, diagnosed with early-stage HER2-positive breast cancer, were retrospectively analyzed in this multicenter study. All patients had received adjuvant treatment with trastuzumab and paclitaxel (TH regimen) between November 2016 and July 2023. The study involved the collection of demographic information, pathological features, and treatment-related details. Overall survival (OS) was defined as the primary study endpoint, while recurrence-free survival (RFS), disease control rate (DCR), and treatment-related neuropathy were evaluated as secondary outcomes. **Results**: The median follow-up time was 70.9 months. The 2-year and 5-year OS rates were 95.3%, and the 5-year RFS rate was 96.8%. No statistically significant differences in OS or RFS were observed in relation to tumor size (T1 vs. T2), hormone receptor status, Ki-67 index, tumor grade, or the use of adjuvant endocrine or radiotherapy (all *p* > 0.05). Neuropathy developed in 53.5% of patients, mostly grade 1. **Conclusions**: Adjuvant TH therapy shows favorable long-term outcomes in early-stage HER2-positive breast cancer.

## Simple Summary

Breast cancer is among the most frequently seen cancers in women, and certain subtypes may grow rapidly and have a higher chance of coming back after treatment. HER2-positive breast cancer is one of these subtypes. Although intensive treatments have significantly improved survival rates, patients with smaller tumors and no lymph node involvement might not require such aggressive approaches. This study evaluated the outcomes of early-stage HER2-positive breast cancer patients who received trastuzumab and paclitaxel following surgery. The treatment was generally well tolerated, with excellent long-term survival and low recurrence observed. Importantly, these results reflect real-world clinical practice rather than being limited to controlled clinical trial settings. This suggests that the therapy is a reliable and safe option for lower-risk patients. The findings may assist physicians in tailoring treatment plans that offer strong protection against cancer recurrence while minimizing potential side effects.

## 1. Introduction

Breast cancer is the most commonly diagnosed cancer and the leading cause of cancer-related mortality among women worldwide. According to the GLOBOCAN 2020 database, more than 2.3 million new cases of breast cancer were diagnosed globally, accounting for approximately 11.7% of all cancer diagnoses and resulting in nearly 685,000 deaths. In Türkiye, breast cancer is the most prevalent malignancy in women, with a rising incidence particularly observed in younger populations over the past decade. This growing burden highlights the urgent need for optimized, risk-adapted treatment strategies in early-stage breast cancer.

Breast cancer is a heterogeneous disease, and treatment decisions must be individualized according to molecular subtypes. For example, HER2-targeted therapies such as trastuzumab have significantly improved outcomes in HER2-positive breast cancer [1], whereas they offer no benefit in triple-negative breast cancer (TNBC), which lacks expression of HER2, estrogen, and progesterone receptors.

Approximately 15–20% of early-stage breast cancers overexpress the human epidermal growth factor receptor 2 (HER2), a biomarker historically linked to increased tumor aggressiveness and recurrence risk [1,2]. While HER2 positivity was once associated with poor prognosis, the introduction of anti-HER2 therapies—particularly trastuzumab—has markedly improved survival outcomes. However, determining the optimal adjuvant therapy for patients with small, node-negative HER2-positive tumors remains a clinical challenge, as the absolute benefit of cytotoxic chemotherapy in this subgroup may be limited and must be weighed against potential toxicity.

Several studies have focused on less intensive treatment strategies as a way to prevent overtreatment. Among these, the Adjuvant Paclitaxel and Trastuzumab (APT) trial notably reported a 3-year invasive disease-free survival (iDFS) rate of 98.7%, achieved with weekly paclitaxel combined with trastuzumab (TH regimen) in patients with node-negative, HER2-positive breast cancer [3]. This evidence has positioned the TH regimen as a preferred adjuvant therapy for carefully selected low-risk patients. Consistent with these results, both the St. Gallen International Consensus and the National Comprehensive Cancer Network (NCCN) guidelines recommend the TH regimen for HER2-positive tumors measuring between 0.5 and 2 cm without nodal involvement [4,5].

Additional studies, such as the ATTEMPT trial, have investigated alternative regimens, including trastuzumab emtansine (T-DM1), which showed a 3-year iDFS rate of 97.8% compared to 93.4% with TH [6]. However, as a phase II study with limited follow-up and statistical power, ATTEMPT has not altered current clinical practice. Importantly, long-term (5–10 year) survival data—essential for guiding adjuvant decisions—remain limited for these newer agents.

Similarly, the phase III ALTTO trial, which compared trastuzumab plus lapatinib with trastuzumab alone in early HER2-positive disease, did not demonstrate an added benefit in disease-free or overall survival, particularly among lower-risk patients [7]. These findings suggest that intensifying therapy beyond trastuzumab may not confer meaningful clinical benefit in node-negative populations.

Despite growing consensus on the efficacy and tolerability of the TH regimen, real-world data remain limited. Clinical trials often enroll highly selected patient cohorts under controlled conditions, which may not reflect the diversity and complexity encountered in routine practice, including patients with comorbidities, older age, or suboptimal treatment adherence.

While randomized controlled trials (RCTs) have established the efficacy of anti-HER2 therapies, it is increasingly recognized that the highly selective nature of these trials may not fully capture the diversity of patients seen in routine clinical practice [8]. In real-world settings, patients may present with comorbidities, varying adherence levels, and broader tumor characteristics that are often underrepresented in RCTs. Therefore, real-world evidence is essential to validate the generalizability of clinical trial results and to guide personalized treatment strategies [9].

In particular, data focusing on patients from specific regional populations, such as Türkiye, are limited in the literature. Regional treatment patterns, genetic profiles, and healthcare access may influence outcomes and safety profiles, underlining the importance of collecting multicenter, real-world data across diverse healthcare settings. Furthermore, while international guidelines currently recommend de-escalation strategies for low-risk HER2-positive breast cancer, real-world confirmation of their long-term efficacy and safety remains an unmet clinical need [4,5,10].

Emerging strategies, such as immunotherapy and molecular profiling, are also gaining attention in early-stage breast cancer. Although immune checkpoint inhibitors (ICIs) are not yet standard in the adjuvant setting for HER2-positive disease, encouraging results from metastatic contexts may open avenues for future application [11]. Furthermore, genomic tools and intrinsic subtype classification (e.g., HER2-enriched subtype via PAM50 assay) may enhance risk stratification and guide personalized treatment approaches.

Against this backdrop, we conducted a multicenter, retrospective study to evaluate the real-world outcomes of adjuvant paclitaxel and trastuzumab (TH) therapy in early-stage, HER2-positive, node-negative breast cancer. Our primary aim was to assess long-term overall survival (OS) and recurrence-free survival (RFS), along with the incidence of treatment-related neuropathy and subgroup-specific outcomes. By applying consistent evaluation criteria such as CTCAE v5.0 and guideline-defined risk stratification [12,13], this study seeks to contribute valuable real-world evidence on the effectiveness, safety, and clinical utility of the TH regimen across a regionally diverse patient population.

## 2. Materials and Methods

### 2.1. Patients and Control Groups

This study was designed as a multicenter, retrospective investigation. Between November 2016 and July 2023, a total of 129 patients aged 18–75 years with early-stage HER2-positive breast cancer were included across participating centers.

To enhance transparency and facilitate understanding of the study design, a flow diagram has been incorporated to visually summarize the patient selection process. This diagram outlines each step, including the total number of patients initially assessed, the application of inclusion and exclusion criteria, and the final cohort analyzed.

Inclusion criteria were as follows:

1. Histopathological diagnosis of invasive breast cancer
2. HER2 positivity confirmed by IHC 3 + or FISH amplification
3. Clinical stage I or II disease
4. Completion of adjuvant therapy after surgery and at least 6 months of follow-up

Exclusion criteria included the following:

1. Stage IV (metastatic) disease at diagnosis
2. HER2-negative tumor profile
3. Incomplete treatment or insufficient follow-up

Clinical information was retrospectively obtained from patient medical records. The evaluated variables included patient age, menopausal status, tumor location (right or left breast), tumor diameter, histological grade, estrogen receptor (ER) and progesterone receptor (PR) status, Ki-67 proliferation index, HER2 expression, lymphatic, vascular, and perineural invasion status, receipt of adjuvant radiotherapy and/or endocrine therapy, presence and severity of treatment-related neuropathy, recurrence occurrence, patient survival status, and length of follow-up.

### 2.2. Study Endpoints

The main outcome measure of this study was overall survival (OS), which was assessed from the date of breast cancer diagnosis until the time of death from any cause or the last available follow-up.

Recurrence-free survival (RFS) served as the secondary outcome and was calculated from the date of diagnosis to the first confirmed recurrence or death, regardless of cause.

In addition to survival outcomes, the frequency and severity of treatment-related neuropathy and other adverse effects were analyzed. The severity of neuropathy was classified using the Common Terminology Criteria for Adverse Events (CTCAE), version 5.0.

### 2.3. Statistical Analysis

Statistical computations were carried out with IBM SPSS Statistics software (version 25.0; IBM Corp., Armonk, NY, USA). Descriptive analyses were applied to outline the initial demographic and clinical profiles of the participants. Categorical variables were expressed as frequencies and percentages (%), while continuous variables were presented as mean and standard deviation (SD), or as median with minimum and maximum values (min–max), depending on the data distribution. Minimum and maximum values were intentionally reported to demonstrate the full range of the data. Interquartile range (IQR) was not calculated for this study.

A *p*-value of less than 0.05 was considered statistically significant. The selection of these statistical methods was based on the type of data and study endpoints: the Kaplan–Meier method and log-rank test were selected due to their robustness in handling censored data and subgroup comparisons. No multivariate analysis was conducted due to the limited number of events (recurrence or death) in the cohort, which would undermine the reliability of a Cox proportional hazards model.

## 3. Results

### 3.1. Patient and Treatment Characteristics

A total of 129 patients participated in the study. The average age was 54.42 (13.01) years, with a median of 54 years and a range from 29 to 83 years. The majority of the patients (79.8%) were younger than 65 years. Postmenopausal status was identified in 59.7% of the cases. Tumor involvement was on the right breast in 45.0% of the patients and on the left breast in 55.0%.

The most common tumor stage was T1c (48.1%), followed by T1b (28.7%), T2 (14.7%), and T1a (8.5%). Nearly half of the tumors were Grade 3 (49.6%), while 42.6% were Grade 2 and 7.8% were Grade 1. Estrogen receptor (ER) positivity was observed in 68.2% of the patients, and progesterone receptor (PR) positivity was noted in 52.7%. All tumors were confirmed as HER2-positive. The Ki-67 index was greater than 20% in 62% of the cases.

Lymphatic invasion was present in 56.6% of the patients, vascular invasion in 23.3%, and perineural invasion in 15.5%. Adjuvant radiotherapy and adjuvant endocrine therapy were administered to 84.5% and 64.3% of the patients, respectively.

Neuropathy was observed in 53.5% of the patients. Among those, 42.0% had Grade 0 neuropathy, 47.8% had Grade 1, and 10.1% had Grade 2 neuropathy.

The recurrence rate was 3.1% (*n* = 4), and the mortality rate was 4.7% (*n* = 6). The mean follow-up duration was 69.05 (34.01) months (median: 70.90 months; range: 5.57–187.47 months). Detailed socio-demographic and clinical characteristics of the study population are presented in Table 1.

**Table 1:** Distribution of socio-demographic and clinical variables.

Variables	n	%
**Age**		
Mean (SD)	54.42 (13.01)	
Median (min-max)	54.00 (29–83)	
≤65	103	79.8
>65	26	20.2
**Menopause**		
Pre	52	40.3
Post	77	59.7
**Breast Laterality**		
Right	58	45.0
Left	71	55.0
**Tcat**		
T1a	11	8.5
T1b	37	28.7
T1c	62	48.1
T2	19	14.7
**Hgrad**		
Grade1	10	7.8
Grade2	55	42.6
Grade3	64	49.6
**ER**		
Negative	41	31.8
Positive	88	68.2
**PR**		
Negative	61	47.3
Positive	68	52.7
**HER2**		
Positive	129	100.0
**Ki67**		
<20	49	38.0
>20	80	62.0
**LI**		
Absent	56	43.4
Present	73	56.6
**VI**		
Absent	99	76.7
Present	30	23.3
**PI**		
Absent	109	84.5
Present	20	15.5
**ADJ-RT**		
Absent	20	15.5
Present	109	84.5
**ADJ-ET**		
Absent	46	35.7
Present	83	64.3
**Neuropathy**		
Absent	60	46.5
Present	69	53.5
**Neuropathy grade**		
Grade0	29	42.0
Grade1	33	47.8
Grade2	7	10.1
**Recurrence**		
Absent	125	96.9
Present	4	3.1
**Mortality**		
Right	123	95.3
Ex	6	4.7
**Follow-up Duration (months)**		
Mean (SD)	69.05 (34.01)	
Median(min-max)	70.90 (5.57–187.47)	

All data are presented as mean (SD), median (minimum–maximum), or absolute number (%) as appropriate. Abbreviations: ER—Estrogen receptor; PR—Progesterone receptor; HER2—Human epidermal growth factor receptor 2; LI—Lymphatic invasion; VI—Vascular invasion; PI—Perineural invasion; ADJ-RT—Adjuvant radiotherapy; ADJ-ET—Adjuvant endocrine therapy.

Neuropathy grades are classified according to the Common Terminology Criteria for Adverse Events (CTCAE).

### 3.2. Survival Outcomes

As presented in Table 2, the overall 2-year and 5-year OS rates were both 95.3%. There were no statistically significant differences in OS based on menopausal status, age, breast laterality, tumor category (Tcat), Ki-67 index, progesterone receptor (PR) status, estrogen receptor (ER) status, vascular invasion (VI), perineural invasion (PI), lymphatic invasion (LI), adjuvant radiotherapy (ADJ-RT), adjuvant endocrine therapy (ADJ-ET), or the presence of neuropathy (all *p* > 0.05).

In Table 3, the 2- and 5-year RFS rates were determined as 96.8%, and the median RFS was not reached during the study period. When evaluated across different subgroups—such as age, tumor laterality, tumor size, tumor grade, estrogen receptor (ER) and progesterone receptor (PR) status, Ki-67 index, lymphatic invasion, use of adjuvant endocrine therapy, and the presence of neuropathy—no statistically significant differences in RFS were identified (all *p* > 0.05).

**Figure 1:**
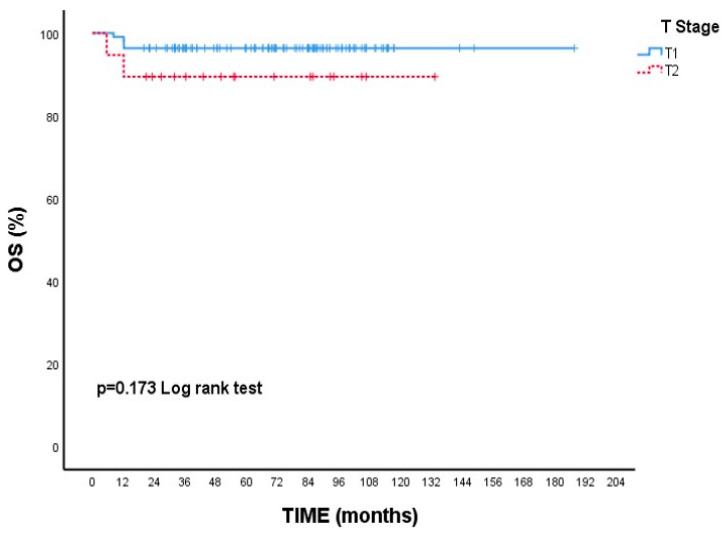
Overall survival (OS) according to T1 and T2 tumor stages: Kaplan–Meier curve. Although patients with T2 tumors showed a numerically lower survival rate, the difference was not statistically significant (log-rank test, *p* = 0.173).

**Figure 2:**
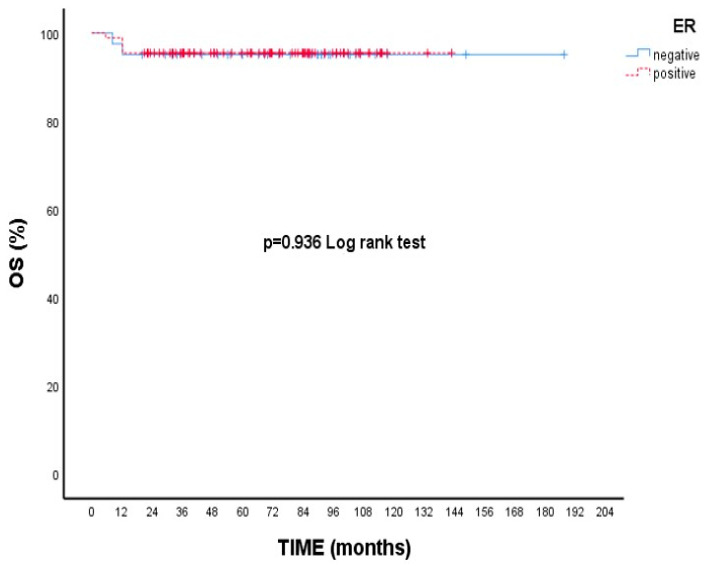
Overall survival (OS) according to estrogen receptor (ER) status: Kaplan–Meier curve. There was no statistically significant difference in overall survival between ER-positive and ER-negative patients (log-rank test, *p* = 0.936).

Although there is no statistical difference, the 5-year survival rate is numerically lower in T2 tumors than in T1 tumors. (89.5% vs. 96.4% *p* = 0.173, Figure 1). Likewise, 5-year OS rates were nearly identical between ER-positive and ER-negative patients (95.5% vs. 95.1%, *p* = 0.936, Figure 2).

**Figure 3:**
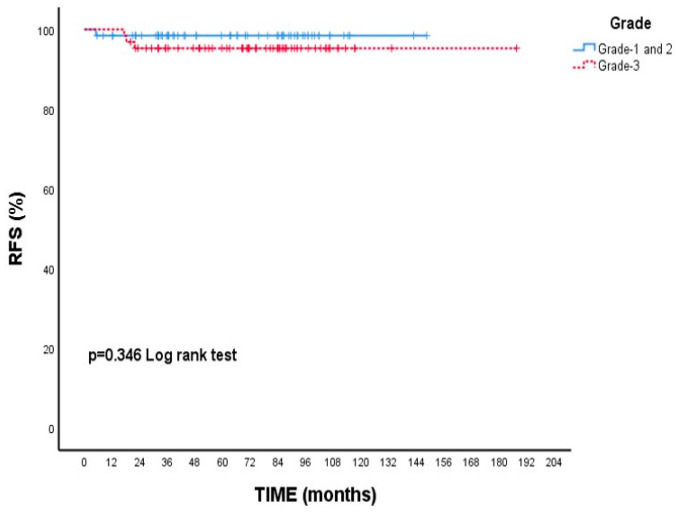
Recurrence-free survival (RFS) according to tumor grade: Kaplan–Meier curve. Although patients with grade 3 tumors had numerically lower RFS compared to those with grade 1–2 tumors, the difference was not statistically significant (log-rank test, *p* = 0.346).

**Figure 4:**
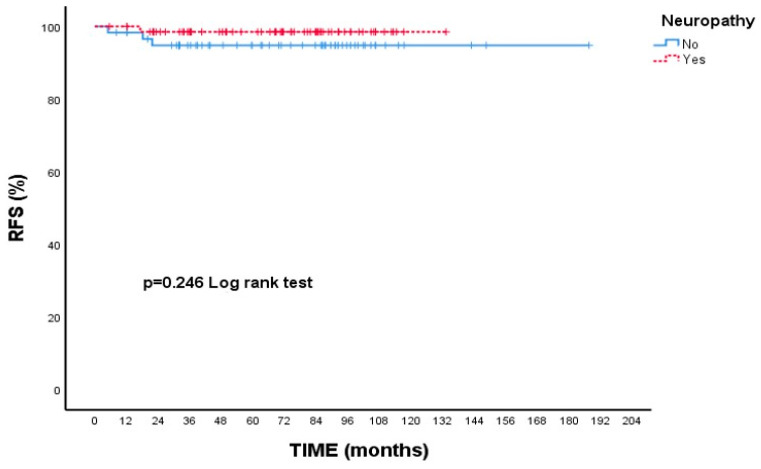
Recurrence-free survival (RFS) according to presence of neuropathy: Kaplan–Meier curve. Although patients with neuropathy showed a numerically higher RFS compared to those without neuropathy, the difference was not statistically significant (log-rank test, *p* = 0.246).

When focusing on RFS, patients with Grade 3 tumors had a 5-year rate of 95.3%, while those with Grade 1–2 tumors had a slightly higher rate of 98.5%; however, this variation was not statistically meaningful (*p* = 0.346, Figure 3). Additionally, patients who experienced neuropathy during treatment showed a higher 5-year RFS (98.5%) compared to those without neuropathy (94.8%), though this difference did not reach statistical significance (*p* = 0.246, Figure 4).

### 3.3. Multivariate Cox Regression Analysis

**Figure 5:**
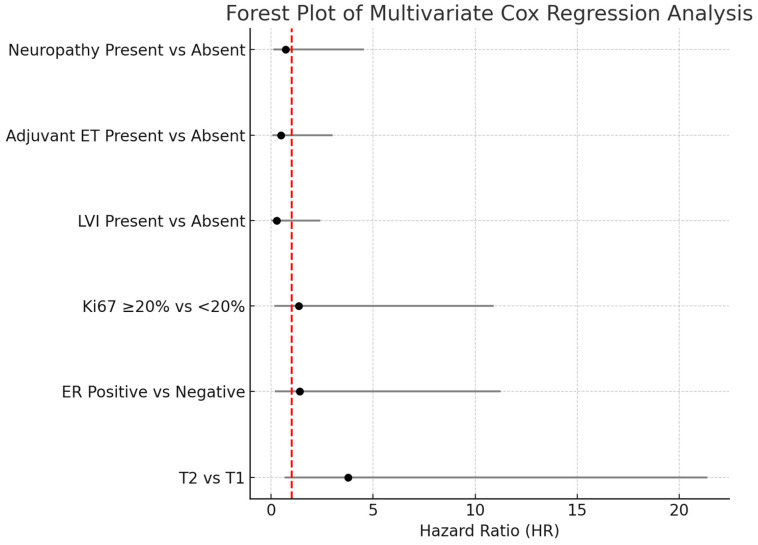
Forest plot of multivariate Cox proportional hazards regression analysis. The horizontal bars represent the 95% confidence intervals for the hazard ratios (HR). Variables with confidence intervals crossing the vertical reference line at HR = 1 were not statistically significant. Abbreviations: HR-Hazard ratio; LVI-Lymphovascular invasion; ER-Estrogen receptor; ET-Endocrine therapy.

As shown in Table 4, T category, ER status, Ki-67, lymphovascular invasion (LVI), adjuvant endocrine therapy (ADJ-ET), and neuropathy variables were included in the multivariate Cox regression model. According to the model results, none of these variables had a statistically significant effect on the risk of death (*p* > 0.05). The results of the multivariate Cox regression analysis are illustrated in Figure 5.

The multivariate Cox regression analysis was summarized using a forest plot to evaluate the impact of various clinical parameters on overall survival. Although the T2 tumor category appeared to be associated with an increased risk of death compared to T1 tumors (HR: 3.78; 95% CI: 0.66–21.37; *p* = 0.132), this association did not reach statistical significance. Similarly, no significant associations were observed for estrogen receptor status, Ki-67 proliferation index, lymphovascular invasion, adjuvant endocrine therapy, or the presence of neuropathy, as all confidence intervals crossed unity and *p*-values were above 0.05. These findings suggest that none of the evaluated clinical variables independently predicted mortality in our cohort.

### 3.4. Safety

Neuropathy was observed in 53.5% of the patients treated with the paclitaxel plus trastuzumab regimen, as shown in Table 5. Among these patients, 42.0% were classified as Grade 0 (asymptomatic or no clinical impact), 47.8% as Grade 1 (mild symptoms), and 10.1% as Grade 2 (moderate symptoms). Importantly, no patients developed Grade 3 or higher neuropathy. Throughout the follow-up period, no cardiotoxicity was detected in any patient. None of the patients discontinued treatment due to neuropathy or other adverse events.

**Table 5:** Adverse events with paclitaxel + trastuzumab.

Adverse Event *	N (%)
Neuropathy (any grade)	69 (53.5)
Neuropathy Grade 0	29 (42.0)
Neuropathy Grade 1	33 (47.8)
Neuropathy Grade 2	7 (10.1)
Cardiotoxicity	0 (0.0)

* Adverse events were categorized and graded as per the Common Terminology Criteria for Adverse Events, version 5.0.

## 4. Discussion

This multicenter study retrospectively evaluated 129 early-stage HER2-positive breast cancer patients. The median follow-up time was 70.9 months, with overall survival (OS) and recurrence-free survival (RFS) rates being generally high. The 2- and 5-year OS rates were 95.3%, and no significant differences were observed based on age, menopausal status, tumor stage, ER/PR status, Ki-67 index, invasion status, adjuvant therapy, or neuropathy. Despite being based on real-world data, the high OS rates demonstrate the successful implementation of effective adjuvant treatment strategies in early-stage disease. These results are consistent with randomized clinical trials reporting 5-year OS rates between 93% and 96% [10].

Unlike the highly selected patient populations included in studies such as APT and ATTEMPT, our cohort reflects broader clinical heterogeneity, including patients with T2 tumors (14.7%), individuals with comorbid conditions, and variability in the administration of endocrine and radiotherapy treatments. Additionally, the inclusion of subgroup analyses focusing on prognostic factors often underreported in randomized controlled trials—such as neuropathy, lymphovascular invasion, and proliferation index—provides a more detailed and nuanced assessment of prognosis.

The 5-year RFS rate observed in our study was 96.8%, which exceeds the 93.3% reported in the APT trial, despite the presence of higher-risk features in our cohort. This finding reinforces the effectiveness and durability of the TH regimen in real-world conditions. 

Regarding estrogen receptor status, no significant difference in OS was found between ER-positive and ER-negative patients. RFS was also not statistically different across any of the variables. Specifically, the 5-year RFS rate was 94.9% in ER-negative and 97.7% in ER-positive patients. In the comparison between T1 and T2 tumors, the RFS rates were 97.2% and 94.1%, respectively. The Ki-67 index did not significantly affect RFS, and no significant difference in RFS was observed between patients who did and did not receive adjuvant endocrine therapy.

Although these differences were not statistically significant, the trends align with previous studies reporting better outcomes in ER-positive, HER2-positive early-stage breast cancer. Both the APT and APHINITY trials have demonstrated that ER-positive cases generally achieve more favorable treatment outcomes [3,14]. These findings support the hypothesis that ER expression in HER2-positive tumors is associated with a less aggressive biological phenotype, potentially contributing to improved long-term disease control.

In the APT trial, approximately 9% of patients had T2 tumors, whereas in our study, this rate was 14.7%, with 3.1% of patients having tumors larger than 3 cm. Unlike the APT trial, which did not report survival analyses based on tumor size, our study conducted a subgroup analysis comparing 5-year RFS rates between T1 and T2 tumors. Although the difference was not statistically significant, our findings contribute valuable real-world evidence, demonstrating that the efficacy of the TH regimen is maintained even in patients with larger tumors.

These findings indicate that most patients remained recurrence-free following adjuvant therapy, with an overall low recurrence risk. The recurrence rate of only 3.1% further confirms the effectiveness of adjuvant anti-HER2 therapy in real-world settings. These results are consistent with clinical trials such as HERA and APT, which report 3–5-year İDFS/RFI rates of 90–95%. While our study showed a recurrence rate of 3.1%, the 3-year recurrence rate reported in the APT trial was 1.3% [3]. It should be noted that the relatively small sample size in our study means that even as few as four recurrences could substantially impact the overall recurrence rate.

The 5-year RFS rate was 95.3% in patients with Grade 3 tumors and 98.5% in those with Grade 1–2 tumors; however, the difference was not statistically significant. Although the APT trial did not report RFS outcomes stratified by histological grade, previous studies have suggested that higher-grade tumors may be associated with a slightly increased risk of recurrence in HER2-positive early breast cancer. Our findings, while not statistically significant, may reflect this biological trend.

In our study, the most common adverse event was neuropathy, which was predominantly mild (Grade 1). Unlike cardiac toxicity, which is a well-known concern with trastuzumab-based therapy [15], neuropathy was more frequently observed in our cohort, likely due to paclitaxel administration. Interestingly, the presence of neuropathy was associated with a higher RFS (98.5% vs. 94.8%), although this difference was not statistically significant (*p* = 0.246). This finding may suggest that neuropathy, often seen in patients receiving more intensive treatment, could indicate a more effective regimen associated with lower recurrence risk. This observation is consistent with findings reported in the literature on taxane-based chemotherapies. Peripheral neuropathy has been associated with pathological complete response (pCR) following paclitaxel treatment in early-stage breast cancer, supporting the notion that neuropathy may serve as a surrogate marker of treatment response [16].

Importantly, our results are largely consistent with the existing literature, and no major contradictions were observed. The only unexpected finding—numerically higher OS in patients with lymphatic invasion—was not statistically significant and is likely related to possible bias and the low event rate. This OS difference may also align with trends seen in patients with T2 tumors and those experiencing treatment-related neuropathy, suggesting that unmeasured confounders or favorable baseline characteristics may have influenced survival outcomes. Overall, our study reinforces current guideline recommendations and provides additional support for the clinical utility of the TH regimen in real-world settings.

Nevertheless, our study has several limitations. Due to its retrospective design, there is potential for selection bias. Additionally, treatment adherence, dose intensity, and toxicity may not have been consistently recorded across all centers. Another limitation is the lack of data on ethnic background. Since the study was conducted in Türkiye, the population is relatively ethnically homogeneous, and no formal data on ethnic subgroups were collected. Future multicenter studies involving more ethnically diverse cohorts are warranted to explore whether treatment efficacy or toxicity may vary by ethnicity.

Despite these limitations, the multicenter design enhances the generalizability of our results. Although the TH regimen has shown excellent outcomes in the adjuvant setting for low-risk, node-negative HER2-positive tumors, it is not routinely used in the neoadjuvant setting. Patients with larger tumors or node-positive disease typically receive more intensive regimens, including dual HER2 blockade and anthracycline–taxane combinations. Therefore, our findings primarily apply to post-surgical adjuvant therapy rather than preoperative treatment approaches [17,18,19,20,21,22].

Compared to the APT study, our study has some important limitations. Our follow-up period is shorter than that of the APT study, which may limit our ability to capture late recurrences. Advanced biological analyses such as molecular biomarkers, HER2DX scoring, PAM50 subtyping, and stromal TIL evaluation were not included in our study. Therefore, our results should be interpreted with caution, especially when considering biological subgroups. Nevertheless, achieving outcomes comparable to, and in some aspects even better than, those of the APT study supports the real-world effectiveness of adjuvant trastuzumab therapy.

In comparison with alternative regimens, the ATEMPT trial compared the TH regimen with T-DM1, reporting 3-year DFS rates of 97.8% in the T-DM1 arm and 93.4% in the TH arm [8]. Considering that ATEMPT was a phase II trial with a relatively short follow-up period, the 5-year RFS rate of 96.8% observed in our study provides robust support for the efficacy of the TH regimen. Additionally, the limited sample size and follow-up duration of the ATEMPT study underscore the need for long-term outcome data. 

The ALTTO study demonstrated that the combination of trastuzumab and lapatinib did not confer superiority over trastuzumab monotherapy [7]. This finding suggests that more aggressive or dual HER2-targeted strategies may not necessarily yield additional benefit. In this context, tolerable and evidence-based protocols such as the TH regimen may represent a suitable approach for patients with low to intermediate risk.

Similarly, the APHINITY trial showed that pertuzumab conferred additional benefit in high-risk patients (e.g., those with node-positive disease), whereas no significant difference was observed in the node-negative subgroup [14]. This supports the notion that single-agent anti-HER2 therapy may be sufficient in node-negative patients, such as those included in our study.

Other real-world data also report favorable outcomes with adjuvant anti-HER2 therapies in patients with low-risk HER2-positive breast cancer [20,23,24,25,26,27,28,29,30].

In conclusion, our study demonstrates that adjuvant trastuzumab therapy in patients with early-stage HER2-positive breast cancer yields outcomes largely consistent with those reported in the APT trial, and may even be superior in certain aspects under real-world conditions. Further validation in larger, long-term studies is needed.

## 5. Conclusions

This multicenter, real-world study demonstrates that adjuvant trastuzumab and paclitaxel (TH) therapy provides excellent long-term outcomes in patients with early-stage, HER2-positive, node-negative breast cancer. The findings confirm the effectiveness of the TH regimen beyond controlled clinical trial settings.

Although subgroup analyses indicated a trend toward lower survival in patients with larger tumors and those with high-grade histology, these differences were not statistically significant. Nevertheless, these observations suggest that closer follow-up and potentially individualized management may be beneficial in these subgroups.

Neuropathy was commonly observed, but the majority of cases were low-grade, and no cardiotoxicity was detected, reinforcing the favorable and manageable safety profile of the TH regimen.

These real-world results are consistent with previously reported clinical trial data and support the continued use of the TH regimen as a standard adjuvant treatment option in low- to intermediate-risk HER2-positive patients, including those who may not be well represented in randomized trials.

Future prospective studies with larger patient populations and longer follow-up periods are warranted to validate these findings and further refine risk-adapted treatment strategies.

## Figures and Tables

**Table 2:** Comparisons of OS among patients.

Variables	2 Years %	5 Years %	Median (95% CI)	*p*
**Overall**	95.3	95.3	- (-)	
**Age**				
≤65	96.1	96.1	- (-)	0.394
>65	92.3	92.3	- (-)	
**Menopause**				
Pre	94.2	94.2	- (-)	0.634
Post	96.1	96.1	- (-)	
**Breast Laterality**				
Right	96.6	96.6	- (-)	0.549
Left	94.4	94.4	- (-)	
**Tcat**				
T1	96.4	96.4	- (-)	0.173
T2	89.5	89.5	- (-)	
**ER**				
Negative	95.1	95.1	- (-)	0.936
Positive	95.5	95.5	- (-)	
**PR**				
Negative	95.1	95.1	- (-)	0.895
Positive	95.6	95.6	- (-)	
**Ki67**				
<20	93.9	93.9	- (-)	0.524
>20	96.3	96.3	- (-)	
**LI**				
Absent	92.9	92.9	- (-)	0.248
Present	97.3	97.3	- (-)	
**VI**				
Absent	97.0	97.0	- (-)	0.110
Present	90.0	90.0	- (-)	
**PI**				
Absent	95.4	95.4	- (-)	0.952
Present	95.0	95.0	- (-)	
**ADJ-RT**				
Absent	90.0	90.0	- (-)	0.192
Present	96.3	96.3	- (-)	
**ADJ-ET**				
Absent	93.5	93.5	- (-)	0.443
Present	96.4	96.4	- (-)	
**Neuropathy**				
Absent	95.0	95.0	- (-)	0.864
Present	95.7	95.7	- (-)	

Overall survival (OS) rates at 2 and 5 years are presented as percentages. The median OS could not be reached due to the low number of events during the follow-up period. Statistical comparisons between subgroups were performed using the Kaplan–Meier method and log-rank test. Abbreviations: ER—Estrogen receptor; PR—Progesterone receptor; LI—Lymphatic invasion; VI—Vascular invasion; PI—Perineural invasion; ADJ-RT—Adjuvant radiotherapy; ADJ-ET—Adjuvant endocrine therapy. A *p*-value < 0.05 was considered statistically significant.

**Table 3:** Comparisons of RFS among patients.

Variables	2 Years %	5 Years %	Median (95% CI)	*p*
**Overall**	96.8	96.8	- (-)	
**Age**				
≤65	97.0	97.0	- (-)	0.776
>65	95.8	95.8	- (-)	
**Breast Laterality**				
Right	96.4	96.4	- (-)	0.857
Left	97.1	97.1	- (-)	
**Tcat**				
T1	97.2	97.2	- (-)	0.519
T2	94.1	94.1	- (-)	
**Hgrade**				
Grade-1 and 2	98.5	98.5	- (-)	0.346
Grade-3	95.3	95.3	- (-)	
**ER**				
Negative	94.9	94.9	- (-)	0.423
Positive	97.7	97.7	- (-)	
**PR**				
Negative	96.6	96.6	- (-)	0.909
Positive	97.0	97.0	- (-)	
**Ki67**				
<20	97.8	97.8	- (-)	0.604
>20	96.1	96.1	- (-)	
**LI**				
Absent	98.1	98.1	- (-)	0.476
Present	95.8	95.8	- (-)	
**ADJ-ET**				
Absent	97.7	97.7	- (-)	0.672
Present	96.3	96.3	- (-)	
**Neuropathy**				
Absent	94.8	94.8	- (-)	0.246
Present	98.5	98.5	- (-)	

Recurrence-free survival (RFS) rates at 2 and 5 years are presented as percentages. The median RFS could not be calculated due to the low number of recurrence events during the follow-up period. Statistical comparisons between subgroups were performed using the Kaplan–Meier method and log-rank test. Abbreviations: ER—Estrogen receptor; PR—Progesterone receptor; LI—Lymphatic invasion; ADJ-ET—Adjuvant endocrine therapy. A *p*-value < 0.05 was considered statistically significant.

**Table 4:** Multivariate Cox regression analysis evaluating the association between clinical variables and risk of death.

Variables	HR (%95 CI)	
**Tcat**		
T1	ref	0.132
T2	3.78 (0.66–21.37)	
**ER**		
Negative	ref	0.747
Positive	1.40 (0.17–11.24)	
**Ki67**		
<20	ref	0.773
>20	1.35 (0.16–10.90)	
**LI**		
Absent	ref	0.243
Present	0.27 (0.03–2.42)	
**ADJ-ET**		
Absent	ref	0.438
Present	0.48 (0.07–3.01)	
**Nöropati**		
Absent	ref	0.721
Present	0.72 (0.11–4.56)	

-2 Log Likelihood: 54,228, *p* = 0.643.

## Data Availability

All data generated or analyzed during this study are included in this published article.

## Abbreviations

**ER**: Estrogen receptor. **HER2**: Human epidermal growth factor receptor 2. **HR**: Hormone receptor. **RFS**: Recurrence-free survival. **PR**: Progesterone receptor. **ADJ-RT**: Adjuvant radiotherapy. **ADJ-ET**: Adjuvant endocrine therapy. LI: Lymphatic invasion. VI: Vascular invasion. PI: Perineural invasion.
